# Supplementary material for: SyNDI: synchronous network data integration framework
Source: BMC Bioinformatics. 2018 Nov 6;19:403. doi: 10.1186/s12859-018-2426-5 (PMC6219086; doi:10.1186/s12859-018-2426-5)
Supplement: Supplementary file 5 — Files of the example presented in Additional file 4. This additional file is a zip package that contains all files used in Additional file 4. Detailed descriptions of each of these files are presented in a README file included in this zip package. (GZ 30411 kb) [file 12859_2018_2426_MOESM5_ESM.gz › SyNDITools/Meme2Fimo/meme_4.9.0_4.tar.gz!/meme_4.9.0/doc/meme-rename.html]

meme-rename


# meme-rename

**Usage:** `meme-rename [options]`

**Description:**

Copy HTML files from MEME Suite programs to files with
distinct names to make it easier to share them:  
 `<dest>/<program>.<last_dir>.html` 
  
where <last\_dir> is the last directory on the path, and
<program> is the name of the MEME Suite program.

**Input:**

The program takes the names of one or more output directories created by
MEME Suite programs such as MEME, CENTRIMO etc.

**Output:**

The program copies the main HTML file to the destination directory, giving
it a name based on the name of the output directory.

**Options:**

- `[<dir>]+`  - full path of MEME Suite program output directory
- `[-d <dest>]`  - destination directory; default: .
- `[-h] - print help message`

**Bugs:** None known.
